# Development of a Recombinant Fusion Vaccine Candidate Against Lethal *Clostridium botulinum* Neurotoxin Types A and B

**DOI:** 10.3390/vaccines13010039

**Published:** 2025-01-06

**Authors:** Eun-Sun Choi, Seong-Wook Pyo, So-Hyeon Kim, Jun-Ho Jeon, Gi-Eun Rhie, Mi-Ran Yun, Hwajung Yi, Yoon-Seok Chung

**Affiliations:** 1 Division of High-Risk Pathogens, Department of Laboratory Diagnosis and Analysis, Korea Disease Control and Prevention Agency, KDCA, Cheongju 28159, Republic of Korea; pearlces86@korea.kr (E.-S.C.); swde29@korea.kr (S.-W.P.); ssonaki82@korea.kr (S.-H.K.); pobee@korea.kr (H.Y.); 2 Division of Infectious Disease Diagnosis, Chungcheong Regional Center for Disease Control and Prevention, Korea Disease Control and Prevention Agency, KDCA, Cheongju 28159, Republic of Korea; jhjeon78@korea.kr; 3 Director General, Center for Vaccine Research, National Institute of Health, Korea Disease Control and Prevention Agency, KDCA, Cheongju 28159, Republic of Korea; 4 Division of Infectious Disease Vaccine Research, Center for Vaccine Research, National Institute of Health, Korea Disease Control and Prevention Agency, KDCA, Cheongju 28159, Republic of Korea; ivareve125@korea.kr

**Keywords:** *Clostridium botulinum*, botulism, neurotoxin, fusion vaccine, receptor binding domain

## Abstract

Background: Botulinum neurotoxins (BoNTs), produced by *Clostridium botulinum*, are potent protein toxins that can cause botulism, which leads to death or neuroparalysis in humans by targeting the nervous system. BoNTs comprise three functional domains: a light-chain enzymatic domain (LC), a heavy-chain translocation domain (HC_N_), and a heavy-chain receptor-binding domain (HC_C_). The HC_C_ domain is critical for binding to neuronal cell membrane receptors and facilitating BoNT internalization via endocytosis. Accordingly, it may serve as a vaccine candidate, inducing anti-BoNT-neutralizing antibodies in animals. Here, we aimed to develop a vaccine capable of simultaneously defending against both BoNT/A and B. Methods: We combined the HC_C_ domains of botulinum neurotoxin type A (BoNT/A) and botulinum neurotoxin type B (BoNT/B) in *Escherichia coli* to produce a recombinant protein (rHC_C_B-L-HC_C_ArHCcB) that offers dual protection against both toxins by inhibiting their receptor binding. To evaluate the efficacy of the vaccine, mice were immunized intramuscularly with rHC_C_B-L-HC_C_A plus alum thrice at 2-week intervals, followed by the assessment of immunogenicity and protective efficacy. Results: The antibody titer in mice immunized with rHC_C_B-L-HC_C_A was significantly higher than that in mice immunized with alum alone, protecting them from the lethal challenges of BoNT/A (10^5^ 50% lethal dose, LD_50_) and B (10^3^ LD_50_). Conclusion: These findings suggest that rHC_C_B-L-HC_C_A may simultaneously be an effective vaccine candidate against BoNT/A and B.

## 1. Introduction

Botulinum neurotoxins (BoNTs), produced by the bacterium *Clostridium botulinum*, are the primary agents responsible for botulism and rank among the most lethal substances known. These toxins are classified into eight serotypes (A–H), each with a similar structure but distinct antigenic properties [1,2,3]. BoNTs are initially synthesized as single polypeptide chains, which are then post-translationally cleaved into dichains consisting of a 100 kDa heavy chain (HC) and a 50 kDa light chain (LC) connected by a disulfide bond. The HC is further subdivided into two functionally independent domains: the N-terminal translocation domain (HC_N_) and the C-terminal receptor-binding domain (HC_C_) [4]. The HC_N_ (~50 kDa) facilitates the transport of the LC across the endosomal membrane, whereas the HC_C_ (~50 kDa) is essential for binding to specific receptors on cholinergic nerve cells. BoNTs achieve high affinity and specificity for neurons by binding to two receptors: gangliosides and one of the synaptic vesicle proteins, either synaptotagmin (Syt) or synaptic vesicle protein 2 (SV2). BoNT/A uses SV2 as its protein receptor, whereas BoNT/B binds to Syt, specifically the Syt-I and Syt-II isoforms [5,6]. After receptor binding, the toxin is internalized into endosomes via receptor-mediated endocytosis. LC acts as a zinc-dependent endoprotease that selectively cleaves three crucial proteins involved in the docking and fusion of acetylcholine-containing synaptic vesicles with the plasma membrane. The inactivation of a synaptosomal-associated protein of 25 kDa, synaptic vesicle-associated membrane protein, or syntaxin by BoNTs prevents acetylcholine release, leading to neuromuscular paralysis. When exposed to BoNTs, early symptoms may include ptosis, ambiopia, blurred vision, dysphonia, and a dry, sore throat. High doses of BoNT can cause respiratory muscle paralysis, leading to dyspnea, respiratory failure, and potentially death [7,8,9].

Botulism can occur naturally following the colonization of the gastrointestinal tract by neurotoxigenic clostridia (infant or intestinal botulism), consumption of contaminated food (foodborne botulism), or anaerobic wound infections (wound botulism) [10]. The Centers for Disease Control and Prevention (CDC) has reported an average of 208 cases annually between 2015 and 2019 [11]. While botulism is rare in South Korea, the Korea Disease Control and Prevention Agency (KDCA) conducts annual diagnostic tests to detect and confirm cases of the disease. From 2003 to 2024, eleven cases of botulism were reported. Of these, eight cases were foodborne botulism, one case was infant botulism, and two cases occurred through an unknown route. Among the eleven patients, serotypes A (three cases) and B (four cases) were the most common, although these cases occurred in 2003 and 2004, respectively [12]. Since 2019, one case of each serotype Ab, Bf, and E has been reported. In the first case of infant botulism, we described the genome of *C. botulinum* type Ab, which contains two different toxin genes [13]. In the USA, where bivalent toxins have been continuously monitored since their discovery in 1976 [14], cases caused by the bivalent strains Ab, Ba, and Bf account for only 2% (30/1514) of all infant botulism cases [15]. However, three-quarters of patients with bivalent strain-induced botulism experienced respiratory failure during hospitalization, suggesting that patients with bivalent toxin may require more urgent treatment.

Pentavalent botulinum toxoid (PBT) is used as an investigational drug by the CDC for military and research personnel exposed to the toxin. However, PBT has not received FDA approval because of numerous shortcomings in its existing form. The development of new-generation recombinant vaccines may alleviate many such problems associated with toxoids [16]. Due to their extreme toxicity, botulinum toxins have been considered prime candidates for bioweapons, prompting the KDCA to research vaccines and treatments for over a decade to prepare for its potential use in terrorism. The threat is increasing worldwide, and South Korea faces heightened risks as a divided nation. Therefore, developing and stockpiling vaccines for military personnel, researchers, and other frontline personnel to prepare them against bioterrorism scenarios as well as natural occurrences is essential. Therefore, this study aimed to develop a vaccine capable of providing simultaneous protection against both BoNT/A and BoNT/B, addressing the critical need for effective countermeasures against bioterrorism and natural outbreaks.

## 2. Materials and Methods

### 2.1. Construction of Synthetic Genes and Cloning

**Figure 1:**
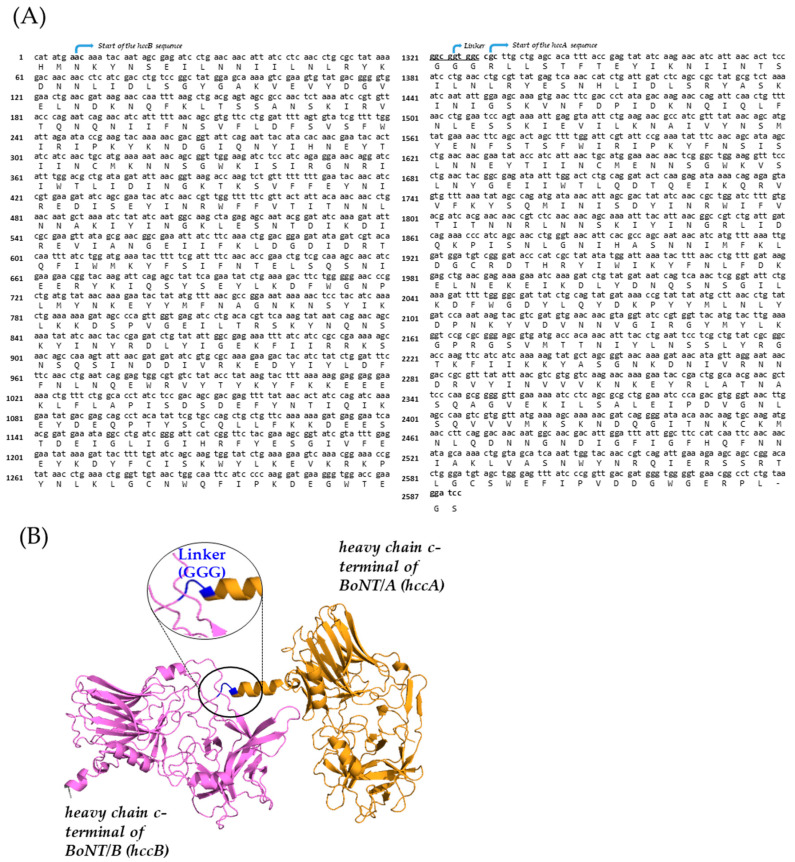
Gene sequence and predicted protein structure of rHCcB-L-HCcA. (**A**) The synthetic gene for rHCcB-L-HCcA expression in *Escherichia coli* is shown with its translated amino acid sequence below the nucleotide sequence. The initial codons of each subunit and the (Gly)3 linker codons are written in bold and marked with arrows. (**B**) Visualization of the three-dimensional structure of the rHCcB-L-HCcA construct, comprising rHCcB (violet), (Gly)3 linker (blue), and rHCcA (orange), predicted using the AlphaFold2 program.

Genes encoding the HCc fragments of BoNT/A (*hccA*, strain 19397; GenBank accession number NC_009697) and BoNT/B (*hccB*, strain Okra; GenBank accession number NC_010516), as well as the fused HCc of BoNT/B and A (hccB-linker-*hccA*), were synthesized by Bioneer Co. (Daejeon, South Korea). A (Gly)3 linker was introduced between the two HCc fragments to facilitate proper folding. After digestion with the restriction enzymes *Nde*I and *Bam*HI, the gene fragments were cloned into the pET19b vector for expression in *E. coli.* The gene sequences and their predicted structures are shown in Figure 1. Gene synthesis was conducted in compliance with the national regulations governing experiments involving genetically modified organisms. All the experimental procedures were performed in a biosafety laboratory and certified by the appropriate authority [17].

### 2.2. Expression and Purification of Recombinant Antigens

Plasmids (pET19b-*hccA*, pET19b-*hccB*, and pET19b-*hccB-L-hccA*) were transformed into different *E. coli* strains to optimize expression: pET19b-*hccA* was transformed into *E. coli* BL21(DE3) RIPL Codon Plus cells, pET19b-*hccB* into *E. coli* BL21(DE3) pLysS cells, and pET19b-*hccB-L-hccA* into *E. coli* BL21(DE3) SoluBL21 cells. The cultures (400 mL for each strain) were grown in Terrific Broth, induced with 0.5 mM isopropyl-β-d-thiogalactopyranoside at an OD_600_ of 0.6 ± 0.1, and incubated at 16 °C for 96 h. The incubated *E. coli* were harvested by centrifugation and resuspended in lysis buffer (50 mM Tris-HCl, 1.5 M NaCl, 1 mM PMSF, 50 µg/mL lysozyme, and 0.1% Triton X100, pH 8.0). The lysed cells were centrifuged to remove the precipitate and filtered through a 0.45 µm disposable filter. The clear supernatant with rHCcA, rHCcB, and rHCcB-L-HCcA antigens was loaded onto a 20 mL column of Ni-NTA superflow (Qiagen, Hilden, Germany), which was equilibrated with 50 mM of Tris-HCl and 1.5 M of NaCl containing 5 mM of imidazole with pH 8.0. The column was washed with washing buffer of different concentrations (50 mM of Tris-HCl and 1.5 M of NaCl containing 60 or 80 mM of imidazole with pH 8.0). Bound rHCcA and rHCcB were eluted from the column using imidazole (50 mM of Tris-HCl and 1.5 M of NaCl containing 100, 200, or 400 mM imidazole with pH 8.0). The bound rHCcB-L-HCcA protein was similarly eluted (50 mM of Tris-HCl and 1.5 M of NaCl containing 250 or 500 mM of imidazole with pH 8.0). Each 5 µL protein sample collected during the purification process was mixed with a loading buffer and loaded onto an SDS-PAGE gel to assess the samples at each step of purification. The eluted proteins were subjected to a final purification step using a fast protein liquid chromatography system (FPLC, Bioneer, Daejeon, South Korea) equipped with a Superdex 200 size-exclusion column (Sigma, Darmstadt, Germany). A 1 mL volume of each eluted protein was loaded onto the column, and fractions containing proteins with a final concentration exceeding 0.5 mg/mL were collected. The eluted rHCcA, rHCcB, and rHCcB-L-HCcA proteins were subsequently dialyzed in 50 mM of sodium citrate buffer (pH 5.5) using a Slide-A-Lyzer Dialysis Cassette (Thermo Scientific, Waltham, MA, USA). The purified proteins were stored at −80 °C until further use.

### 2.3. Identification of Antigens

The BiolineTM Bioterror Pathogens and Toxins Test Kit (Abbott, Seoul, South Korea) was used to verify the identity of the purified antigens. This lateral flow assay, designed for the rapid detection of bioterrorism agents, was adapted to confirm the presence of the BoNT/A and B antigens. The strips were coated with antibodies specific for BoNT/A and B and designed to allow differential detection of the two toxin types on a single strip. Purified rHCcA, rHCcB, and rHCcB-L-HCcA antigens were mixed with the sample diluent, added to the cassette wells, and absorbed. The results were documented after a 30 min incubation.

### 2.4. Preparation of Toxins

BoNT/A was harvested from *C. botulinum* strain ATCC19397 following a 6-day anaerobic culture as described previously [18,19]. BoNT/B complex was purchased from List Bio Laboratory Inc. (Campbell, CA, USA). The potencies of the toxins were determined using standard mouse bioassays with established LD_50_ values for BoNT/A and B. The LD_50_ values of BoNT/A and B in mice were confirmed to be 0.1 ng and 0.18 ng, respectively.

### 2.5. Vaccination and Challenge of Mice

BALB/c mice (female, 5–6 weeks old, KOATECH, South Korea) were immunized with the purified antigens adsorbed onto 0.2% aluminum hydroxide (Al(OH)_3_). A group of five mice was vaccinated intramuscularly at 0, 2, and 4 weeks with 1 µg of rHCcA, rHCcB, rHCcB-L-HCcA, or an antigen mixture, which combined 1 µg of rHCcA and 1 µg of rHCcB, all mixed with alum (25 µg) in a total volume of 0.2 mL. Mice injected with PBS or alum were used as negative controls. Two weeks after the last vaccination, mice were challenged with a 10^5^ LD_50_ dose of BoNT/A or 10^3^ LD_50_ dose of BoNT/B by intraperitoneal injection and observed for 14 days. All animal procedures were approved by the Institutional Animal Care and Use Committee of KDCA (KDCA-IACUC-046-17-2A, 2017).

### 2.6. Measurement of Serum Antibody Titers

Serum samples collected at specified time points were analyzed for antigen-specific antibodies using indirect ELISA. Briefly, ELISA plates (Corning Incorporated, Corning, NY, USA) were coated with an optimal concentration of purified rHCcA or rHCcB (1 µg/mL in bicarbonate, 100 µL/well) overnight at 4 °C. Plates were washed with PBS containing 0.05% Tween 20 (PBST) using a Plate Washer (BioTek, Agilent, CA, USA). The plates were blocked for 1 h with 5% skimmed milk at 37 °C, then washed as described above. Serum samples were initially diluted 1:50, followed by five-fold serial dilutions (1:50–1:3,906,250), and incubated for 2 h at 37 °C. After washing with PBST, a 1:5000 dilution of anti-mouse IgG-HRP (Jackson ImmunoResearch Inc., West Grove, PA, USA) was incubated for 1 h at 37 °C. After washing with PBST, TMB substrate was added to each well and incubated for 10 min at room temperature (25 °C). The colorimetric reaction was stopped with 2 N sulfuric acid, and absorbance at 450 nm was determined using a microplate reader (Molecular Devices, San Jose, CA, USA).

### 2.7. Statistical Analysis

Statistical significance across groups was assessed using one-way analysis of variance (ANOVA), followed by the Kruskal–Wallis test and then Dunn’s multiple comparison test to identify specific group differences. All analyses were conducted using GraphPad Prism 5 (GraphPad, La Jolla, CA, USA). The Kruskal–Wallis test was selected to evaluate differences among groups due to its suitability for non-parametric data. Post-hoc tests, specifically Dunn’s multiple comparison tests, were applied to control for type 1 error in multiple comparisons. Statistical significance was defined as *p* < 0.05. Asterisks (*) in the figures indicate p-values less than 0.05, and double asterisks (**) indicate *p*-values less than 0.01, denoting significant differences between groups. Data are presented as mean ± standard deviation (SD) unless otherwise noted.

## 3. Results

### 3.1. Protein Expression and Verification

**Figure 2:**
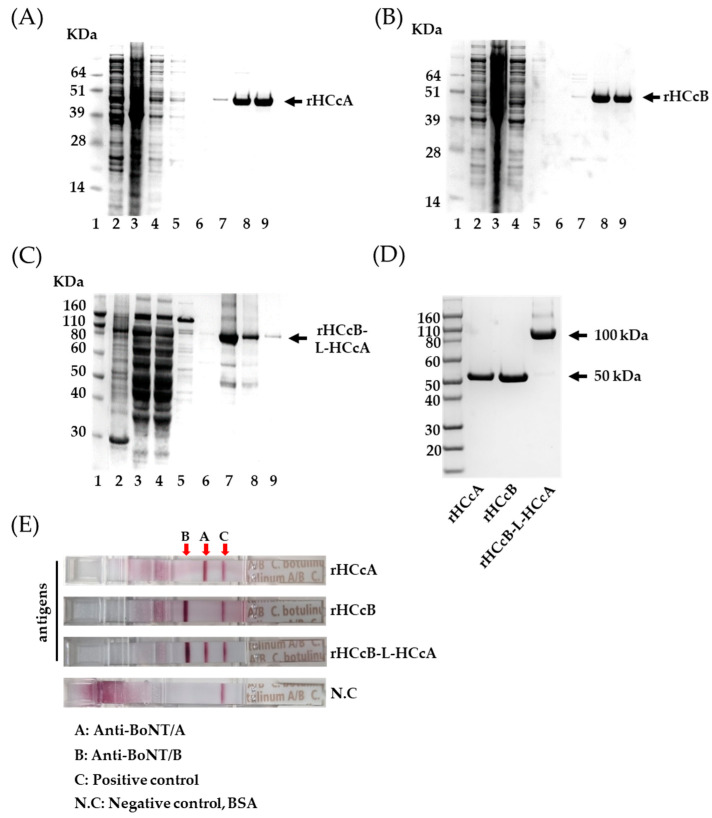
Purification and verification of protein from *E. coli* BL21(DE3) with pET19b-*hccA*, pET19b-*hcB*, and pET19b-*hccB-L-hccA*. (**A**–**C**) SDS-PAGE analysis of samples from the purification process, showing molecular mass markers (Lane 1), soluble cell fraction (Lane 2), insoluble cell fraction (Lane 3), flow-through from the soluble cell fraction in the Ni^2+^ column (Lane 4), wash fractions (Lanes 5–6), and eluted protein (Lanes 7–9). (**D**) SDS-PAGE analysis of purified proteins rHCcA, rHCcB, and rHCcB-L-HCcA, showing expected molecular weights of ~50 kDa and ~100 kDa. (**E**) Verification of purified proteins using an Antigen Rapid Detection Test Kit (Abbott) based on a lateral flow assay.

Codon optimization was employed to facilitate the high-level expression of recombinant BoNT/A HC_C_ (rHCcA), BoNT/B HCc (rHCcB), and rHCcB-L-HCcA. Synthetic genes for *hccA*, *hccB*, and *hccB-L-hccA* were engineered to target the C-terminal fragment of the heavy chain in BoNT/A and B, as illustrated in Figure 1A. To ensure proper folding of each HCc domain, a three-glycine linker was incorporated between the *hccB* and *hccA* segments. The recombinant proteins (rHCcA, HcB, and rHCcB-L-HCcA) were purified from 1 L of the culture using a Ni^2+^ affinity chromatography column. Each purified protein fraction was verified using SDS-PAGE. The results confirmed that the rHCcA and rHCcB proteins were approximately 50 kDa (Figure 2A,B), whereas the rHCcB-L-HCcA fusion protein was approximately 100 kDa (Figure 2C). Thus, the rHCcB-L-HCcA fusion protein exhibited a higher molecular weight owing to the synthesis of rHCcA and rHCcB. To further enhance protein purity, the eluted fractions were subjected to additional purification steps using an FPLC system with Superdex200 column, and the proteins were analyzed by SDS-PAGE (Figure 2D). The results showed that all three proteins collected from the elution buffer possessed a molecular size similar to the 50 kDa of rHCcA and rHCcB proteins and 100 kDa of rHCcB-L-HCcA fusion protein, with high purity. The next experiment was conducted to verify the specific binding of each purified protein antigen with its respective antibody, using a Bioline™ Bioterror Pathogens and Toxins Test Kit developed in previous research to prepare for biological terrorism scenarios. This rapid antigen detection kit features membrane strips coated with specific polyclonal antibodies against BoNT/A and B, enabling the simultaneous detection of these antigens. The assay results revealed that rHCcA and rHCcB were specifically bound to anti-BoNT/A and B antibodies, respectively, which manifested as purple bands. Remarkably, the rHCcB-L-HCcA fusion protein bound to both antibody sets and displayed two distinct purple bands (Figure 2E). These results confirmed that rHCcA, rHCcB, and rHCcB-L-HCcA were specifically bound to their respective antibodies, demonstrating that each protein exhibited high specificity for its intended target.

### 3.2. Immunogenicity and Protective Efficacy

**Figure 3:**
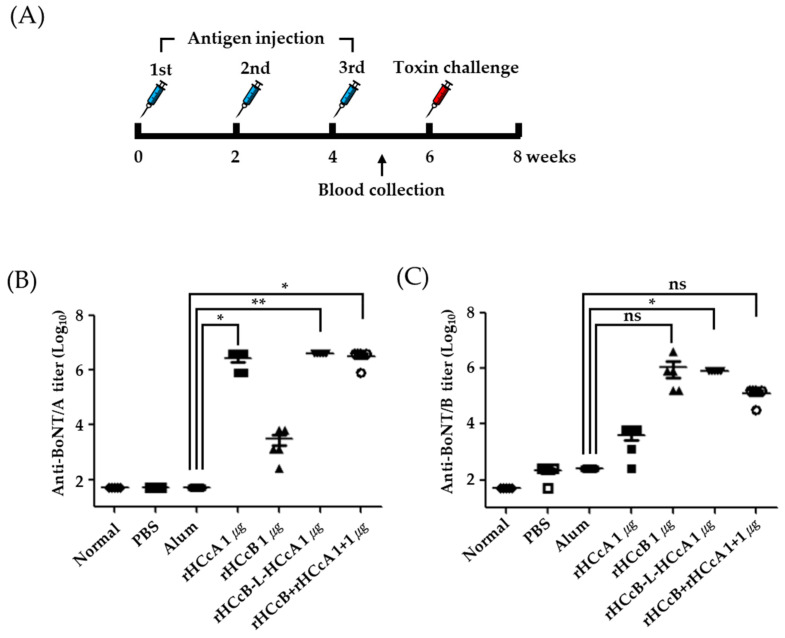
Antibody responses in mice after vaccination with rHCcA, rHCcB, rHCcB-L-HCcA, or a mixture of rHCcB and rHCcA antigens. (**A**) Outline of the immunization study to assess antibody responses post-vaccination in BALB/c mice. (**B**,**C**) Evaluation of antibody response in mice injected with PBS, alum, rHCcA, rHCcB, rHCcB-L-HCcA, or antigen mixture, tested individually using ELISA. The median values for each group are depicted with a bar. Significance levels are indicated as * *p* < 0.05, ** *p* < 0.01; ns indicates no significant difference. Each point on the graph represents an individual mouse.

In the next experiment, we evaluated the immunogenicity induced by the purified antigens (rHCcA, rHCcB, and rHCcB-L-HCcA) and an antigen mixture (rHCcA and rHCcB). BALB/c mice (n = 5/group) were immunized with each antigen mixed with alum (25 µg) thrice at 2-week intervals, as depicted in Figure 3A. One week after the third immunization, blood was collected from the facial veins of mice and separated into serum samples. Serum was then used to determine the binding antibody titers for each antigen via ELISA using plates coated with rHCcA and rHCcB antigens. The experimental results showed that anti-BoNT/A-specific titers in mice immunized with rHCcA, rHCcB-L-HCcA, and antigen mixture were significantly higher than those in the negative controls (mice immunized with alum alone), with all three antigens showing statistical significance (*p* < 0.05; Figure 3B). The results shown in Figure 3C indicate that the anti-BoNT/B-specific titers in mice immunized with rHCcB-L-HCcA were significantly higher (*p* < 0.05) than those in the negative control group. While the rHCcB and antigen mixture groups did not demonstrate statistical significance, it is noteworthy that three out of five mice in the rHCcB group exhibited antibody titers comparable to or exceeding those observed antibody titers comparable to or exceeding those observed in the rHCcB-L-HCcA group (Figure 3C). These results suggest that rHCcA and rHCcB-L-HCcA antigens have the potential to act as vaccines to protect against BoNT/A, with a strong immunogenic response. While the rHCcB antigen exhibited variability among mice, leading to statistically non-significant outcomes, this may be attributed to the small sample size used in the experiment, which could have limited the detection of significant differences. Notably, rHCcB-L-HCcA displayed consistent and robust immunogenicity, indicating its potential to serve as a vaccine candidate for simultaneous protection against both BoNT/A and BoNT/B.

In subsequent experiments, to evaluate the protective efficacy of each antigen, 10^5^ 50% lethal dose (LD_50_) of BoNT/A (equivalent to 10 µg per mouse) and 10^3^ LD_50_ of BoNT/B (equivalent to 0.18 µg per mouse) were injected intraperitoneally into the immunized mice, which were then observed for 2 weeks. As shown in Table 1, the groups immunized with rHCcA, rHCcB-L-HCcA, and antigen mixture exhibited 100% survival against 10^5^ LD_50_ of BoNT/A. Similarly, groups immunized with rHCcB, rHCcB-L-HCcA, and antigen mixture showed 100% survival against 10^3^ LD_50_ of BoNT/B. Conversely, the groups immunized with single antigens rHCcA or rHCcB did not survive when challenged with BoNT/B or BoNT/A, respectively. Through this experiment, we confirmed that there were no significant differences in the ability of single antigens, fusion antigens, and antigen mixtures to protect against BoNTs.

We conducted additional studies to evaluate the protective efficacy when mice were simultaneously challenged with BoNT/A and B. Four mice were immunized with either the rHCcB-L-HCcA antigen or a mixture of rHCcA and rHCcB, with each mouse receiving a 5 µg dose mixed with alum (25 µg) and administered intramuscularly at 2-week intervals for a total of three doses. Two weeks after the final immunization, the mice were challenged intraperitoneally with a mixture of 10^3^ LD_50_ of BoNT/A and 10^3^ LD_50_ of BoNT/B, as well as a mixture of 10^4^ LD_50_ of BoNT/A and 10^4^ LD_50_ of BoNT/B, and were observed for two weeks. The results showed that the group immunized with the rHCcB-L-HCcA antigen had a 100% survival rate when challenged with 10^3^ LD_50_ of BoNT/A + BoNT/B, whereas, in the mixture group, one out of four mice died, resulting in a 75% survival rate. However, both groups succumbed to the challenge with 10^4^ LD_50_ of BoNT/A + BoNT/B (Table 2). These experimental results highlight the unique advantage of the rHCcB-L-HCcA antigen, which demonstrates protective efficacy comparable to single antigens. Additionally, the ability of rHCcB-L-HCcA to protect against both BoNT/A and B simultaneously at lower doses than the antigen mixture suggests a significant advantage as a potential vaccine candidate.

## 4. Discussion

Botulism is a rare disease worldwide; however, in South Korea—a divided nation with potential bioterrorism risks—the development of a vaccine as part of preparedness measures is crucial. Given the potential use of botulinum toxins in bioterrorism and the absence of licensed vaccines, developing an effective dual-protection vaccine remains a pressing global priority. Vaccination was previously recommended only for high-risk groups, including healthcare providers, researchers, first responders, and military personnel at risk of exposure to BoNTs. In 2011, however, the CDC discontinued the investigational PBT (ABCDE) vaccine due to its limited efficacy. As a result, no licensed vaccine is currently available to prevent intoxication caused by the botulinum serotypes most commonly associated with human diseases, namely serotypes A, B, E, and F [20,21]. While post-exposure administration of antitoxins can effectively treat rare cases of life-threatening botulism, these therapies are insufficient for protecting large populations during a potential bioterrorism event involving the dissemination of BoNTs [22]. Thus, there is an urgent need for the proactive development of new therapies and vaccination strategies to mitigate the risk of botulinum neurotoxin intoxication.

The objective of this study was to develop a fusion vaccine capable of simultaneously protecting against botulinum toxin serotypes A and B, with the aim of establishing independent domestic vaccine production. To achieve this, we designed a recombinant fusion protein antigen by combining the heavy chain C-terminal domains of BoNT/A and B. The fusion protein, named rHCcB-L-HCcA, incorporates a glycine linker ((Gly)3) to optimize protein folding, enhance production yield, and improve antibody accessibility (Figure 1). This design aims to overcome common challenges in protein misfolding and low production efficiency often encountered in vaccine development [23,24,25]. The synthetic genes *hccB*, *hccA*, and *hccB-L-hccA* were cloned into the pET19b expression vector, and each plasmid was transformed into *E. coli* BL21(DE3) RIPL Codon Plus, pLysS, and SoluBL21, respectively. Following protein expression, the rHCcA, rHCcB, and rHCcB-L-HCcA antigens were purified using a Ni^2+^ column. We attempted to express a synthetic gene with *hccA* placed upstream, *hccA-L-hccB*, by cloning the pET19b-*hccA-L-hccB* plasmid into *E. coli* BL21(DE3) SoluBL21; however, a pure protein was not obtained. Therefore, we selected the rHCcB-L-HCcA fusion protein as a vaccine candidate against BoNT/A and B and evaluated its efficacy.

The final purified proteins were obtained in sufficient quantities (0.5–1 mg) for experimental use. SDS-PAGE analysis confirmed the purity of each protein, revealing single antigen bands at the expected molecular weights: 50 kDa for the individual proteins and 100 kDa for the fusion protein (Figure 2A–D). The purified proteins (rHCcA, rHCcB, and rHCcB-L-HCcA) were further tested for their reactivity using the BiolineTM Bioterror Pathogens and Toxin Test Kit, enabling the detection of immune reactions with anti-BoNT/A or anti-BoNT/B antibodies coated in the kit. The results confirmed that the rHCcA, rHCcB, and rHCcB-L-HCcA proteins specifically bound to anti-BoNT/A, anti-BoNT/B, and both anti-BoNT/A and anti-BoNT/B simultaneously, respectively (Figure 2E). These results not only demonstrate the successful purification of the antigens but also suggest the potential for generating antibodies specifically targeting each single antigen.

The purified protein antigens (rHCcA, rHCcB, and rHCcB-L-HCcA), each at a dose of 1 µg, along with a mixed antigen containing 1 µg each of rHCcB and rHCcA, were administered intramuscularly with 25 µg of alum to groups of five mice. Immunizations were performed three times at 2-week intervals. As a negative control, phosphate-buffered saline (PBS) as the antigen diluent, combined with 25 µg of alum, was administered under the same conditions as the antigens (Figure 3A). To assess the immunogenicity of the antigen–antibody response, blood samples were collected from the mice one week after the final immunization. The serum was analyzed using ELISA, and the antibody titers for each antigen were logarithmically transformed and statistically evaluated using GraphPad Prism 5. The results shown in Figure 3B indicate that the antigens rHCcA, rHCcB-L-HCcA, and the antigen mixture elicited significantly higher antibody titers when bound to coated rHCcA compared to alum alone. This finding suggests a strong potential for protection against BoNT/A. Among these, the fusion protein rHCcB-L-HCcA demonstrated the highest significance, further emphasizing its promise as a viable vaccine candidate.

Figure 3C shows that both the rHCcB and rHCcB-L-HCcA fusion protein antigens elicited high antibody titers. However, the antigens rHCcB and the antigen mixture did not show statistically significant differences compared to alum. The antibody titer results revealed considerable variability among the five mice in the rHCcB-immunized group, leading to a large standard deviation. One study reported that a small amount of antibodies targeting the HN domain of BoNT/A could block critical active sites of the toxin [26]. This suggests that the protective efficacy observed in certain antigen groups, despite low immunogenicity, may result from functional mechanisms independent of antibody titers. It emphasizes that the structural or functional properties of antigens play a significant role in determining protective efficacy. Furthermore, even with low titers, existing antibodies may act as neutralizing antibodies, effectively targeting and neutralizing the toxin’s active sites [27]. The slightly elevated antibody titer observed in the alum group may have contributed to the lack of statistically significant differences when compared to rHCcB. Another consideration is the heterogeneity often observed among mice in immunization experiments. Small group sizes, such as five mice, can lead to considerable variability in results. To mitigate this issue, future experiments will increase the group size to at least ten mice per group, ensuring more robust statistical reliability.

Despite these challenges, the rHCcB-L-HCcA-immunized group showed statistically significant differences compared to the alum group. These findings strongly suggest that the rHCcB-L-HCcA antigen has dual protective potential against both BoNT/A and BoNT/B, further reinforcing its promise as an effective vaccine candidate.

In the protection assays, mice immunized with each antigen were intraperitoneally challenged with 10^5^ LD50 (10 µg) of BoNT/A and 10^3^ LD50 (0.18 µg) of BoNT/B. The results showed that all mice in the negative control groups (PBS and Alum) succumbed to the toxin challenge. In contrast, mice immunized with rHCcA, rHCcB-L-HCcA, and the antigen mixture demonstrated 100% survival, as expected due to their strong protective efficacy against BoNT/A. Furthermore, mice immunized with rHCcB, rHCcB-L-HCcA, and the antigen mixture also achieved 100% survival against BoNT/B despite exhibiting low antibody titers. As previously mentioned, the results suggest that rHCcB or the antigen mixture, despite having low antibody titers, may effectively block critical active sites of BoNT/B. It is also possible that the antigens directly bind to the toxin, thereby neutralizing its activity. These findings from the protection assays support the potential of the rHCcB-L-HCcA antigen as a promising vaccine candidate capable of providing strong protective efficacy against both BoNT/A and BoNT/B. Furthermore, they highlight the importance of the functional properties of antigens in determining protective efficacy. To investigate whether increasing the antigen dose could enhance antibody titers and protective efficacy, the concentrations of rHCcB, rHCcB-L-HCcA, and the antigen mixture were increased to 5 µg and 10 µg. These antigens were mixed with 25 µg of Alum and administered intramuscularly to mice at 2-week intervals for three immunizations. After the final immunization, blood samples were collected to assess antibody titers and protective efficacy. The experimental results showed that increasing the antigen dose to 10 µg resulted in significant differences in antibody titers for the rHCcB and rHCcB-L-HCcA groups compared to the Alum group (Figure S1). However, despite the increased dose and significant differences in antibody titers, mice in all antigen groups succumbed to a higher toxin challenge of 10⁴ LD50 BoNT/B (1.8 µg) (Table S1). These findings suggest that increasing the antigen dose alone is insufficient to achieve protective efficacy against BoNT/B. The literature also suggests that the lack of protective efficacy, despite high immunogenicity in some antigen groups, can be attributed to glycosylation [28]. Glycosylated forms of BoNT/B(HC) and TeNT(HC) failed to induce protective immunity, while their protective capabilities were restored after undergoing deglycosylation. This indicates that glycosylation can alter the immunogenicity of protein antigens. In this study, we plan to investigate the immunogenicity and protective efficacy of rHCcB after subjecting it to deglycosylation to further assess its potential.

Finally, the protective efficacy of the rHCcB-L-HCcA antigen and the antigen mixture was evaluated against simultaneous challenges with BoNT/A and BoNT/B. The results showed that mice immunized with the rHCcB-L-HCcA antigen survived a mixture of 10^3^ LD50 of BoNT/A and BoNT/B but succumbed to a higher dose of 10^4^ LD50 of the same toxins. In contrast, mice immunized with the antigen mixture exhibited a 75% survival rate against 10^3^ LD50 of BoNT/A and BoNT/B but did not survive the higher toxin dose (Table 2). According to the Supplementary Data, both the rHCcB-L-HCcA antigen and the antigen mixture elicited high antibody titers (Figure S1). However, despite the elevated antibody titers, both groups were only able to protect mice against up to 10^3^ LD50 of BoNT/A and BoNT/B. The exact cause of this limitation remains unclear, but as previously mentioned, the structural or functional properties of the antigens may play a crucial role in determining protective efficacy. It is necessary to analyze antigen-antibody interactions, increase the size of the mouse groups, and evaluate immune responses under various conditions to improve the reliability of the results and clarify the defense mechanisms against BoNT/B. Nonetheless, the rHCcB-L-HCcA antigen is confirmed to be a strong and promising vaccine candidate capable of protecting against 10⁵ LD50 of BoNT/A and 10^3^ LD50 of BoNT/B. These findings not only highlight the potential of the rHCcB-L-HCcA antigen but also provide a foundation for further investigations to optimize its protective efficacy and broaden its application as a dual-protection vaccine against botulinum neurotoxins.

## 5. Conclusions

This study demonstrated that the rHCcB-L-HCcA fusion protein is a promising vaccine candidate capable of providing dual protection against BoNT/A and BoNT/B. The antigen showed strong immunogenicity and protective efficacy against BoNT/A, while the protective efficacy against BoNT/B was achieved despite low antibody titers, highlighting the potential role of functional mechanisms beyond antibody titers. However, the limited protection observed at higher toxin concentrations suggests the need for further optimization. Future research should focus on improving the protective efficacy against BoNT/B through antigen modifications and investigating glycosylation effects and antigen-antibody interactions. These findings provide valuable insights for the development of effective vaccines against botulinum neurotoxins.

## Figures and Tables

**Table 1:** Survival rate of mice immunized ^1^ with antigen following challenge with BoNT/A or BoNT/B.

Antigens	Dose	BoNT/A (10^5^ LD_50_)	BoNT/B (10^3^ LD_50_)
		% (Number of Survivors/Total)	
Alum	25 µg	0 (0/5)	0 (0/5)
rHCcA	1 µg	100 (5/5)	0 (0/5)
rHCcB	1 µg	0 (0/5)	100 (5/5)
rHCcB-L-HCcA	1 µg	100 (5/5)	100 (5/5)
rHCcA + rHCcB	1 µg + 1 µg	100 (5/5)	100 (5/5)

^1^ Mice were immunized thrice at weeks 0, 2, and 4 with 1 µg of rHCcA, rHCcB, or rHCcB-L-HCcA, each mixed with alum as an adjuvant to enhance the immune response. The antigen mixture (rHCcA + rHCcB) was prepared in the same manner as other antigens by combining 1 µg of rHCcA and 1 µg of rHCcB, then mixing with alum before administration to mice. Four weeks after the final booster immunization, mice were challenged with 10^5^ LD_50_ of BoNT/A and 10^3^ LD_50_ of BoNT/B. Challenge numbers indicate the survival rate as the number of surviving mice out of the total number of mice in each group.

**Table 2:** Protective efficacy of rHCcB-L-HCcA and antigen mixture against combined BoNT/A and B exposure in mice ^1^.

Antigens	Dose	BoNT/A (10^3^ LD_50_) + BoNT/B (10^3^ LD_50_)	BoNT/A (10^4^ LD_50_) + BoNT/B (10^4^ LD_50_)
		% (Number of Survivors/Total)	
Alum	25 µg	0 (0/4)	0 (0/4)
rHCcB-L-HCcA	5 µg	100 (4/4)	0 (0/4)
rHCcA + rHCcB	5 µg + 5 µg	75 (3/4)	0 (0/4)

^1^ Mice were immunized with three intramuscular injections of 5 µg of the rHCcB-L-HCcA or antigen mixture, prepared by combining 5 µg of rHCcA and 5 µg of rHCcB per injection, administered at 2-week intervals. Following immunization, mice were challenged intraperitoneally with a mixture of BoNT/A and BoNT/B at doses of 10^3^ LD_50_ and 10^4^ LD_50_.

## Data Availability

Data are contained within the article.

## Supplementary Materials

The following supporting information can be downloaded at https://www.mdpi.com/article/10.3390/vaccines13010039/s1. Table S1: Protective efficacy of rHCcB and rHCcB-L-HCcA antigens against high-concentration BoNT/B exposure in mice. Figure S1: Evaluation of antibody response in mice injected with PBS, alum, rHCcB, rHCcB-L-HCcA, or antigen mixture, tested individually using ELISA.
