# Supplementary material for: Development of a Recombinant Fusion Vaccine Candidate Against Lethal Clostridium botulinum Neurotoxin Types A and B
Source: Vaccines (Basel). 2025 Jan 6;13(1):39. doi: 10.3390/vaccines13010039 (PMC11769440; doi:10.3390/vaccines13010039)

**Supplement Table S1.** Protective efficacy of rHCcB and rHCcB-L-HCcA antigens against high-concentration BoNT/B exposure in mice.

| Antigens | Dose | BoNT/B ($10^4$ $LD_{50}$) |
|---|---|---|
| | | % (Number of survivors/total) |
| Alum | 25 µg | 0 (0/4) |
| rHCcB | 5 µg | 0 (0/4) |
| rHCcB | 10 µg | 0 (0/4) |
| rHCcB-L-HCcA | 5 µg | 0 (0/4) |
| rHCcB-L-HCcA | 10 µg | 0 (0/4) |
| rHCcA+rHCcA | 5 µg + 5 µg | 0 (0/4) |

**Supplement Figure S1.** Evaluation of antibody response in mice injected with PBS, alum, rHCcB, rHCcB-L-HCcA, or mixture antigens, tested individually using ELISA. Median values for each group are depicted with a bar. Significance levels are indicated as *p < 0.05, **p < 0.01. Each point on the graph represents an individual mouse. The rHCcB and rHCcB-L-HCcA antigens were tested at two concentrations, 5 µg and 10 µg, while the mixture antigen was prepared by combining 5 µg each of rHCcA and rHCcB for immunization. ELISA was performed by coating with rHCcB.

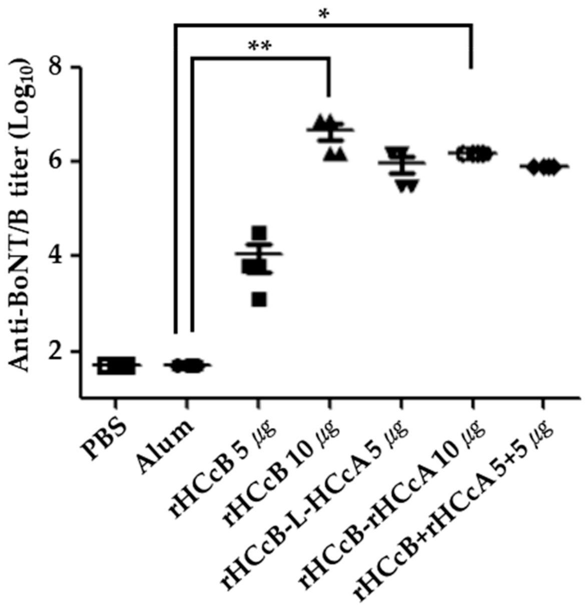

Supplement: Supplementary file 1 [file vaccines-13-00039-s001.zip › vaccines-3371928-supplementary.pdf]
